# Supplementary material for: Kinesin-like motor protein KIF23 maintains neural stem and progenitor cell pools in the developing cortex
Source: EMBO J. 2024 Dec 4;44(2):331–55. doi: 10.1038/s44318-024-00327-7 (PMC11729872; doi:10.1038/s44318-024-00327-7)
Supplement: Supplementary file 1 — Appendix [file 44318_2024_327_MOESM1_ESM.pdf]

Appendix

Kinesin-like motor protein KIF23 maintains neural stem and progenitor cell pools in the developing cortex

Sharmin Naher[1, 2], Kenji Iemura[3], Satoshi Miyashita[4], Mikio Hoshino[4], Kozo Tanaka[3], Shinsuke Niwa[5, 6], Jin-Wu Tsai[7, 8, 9], Takako Kikkawa[2]*, Noriko Osumi[1, 2]*

Appendix Table of Contents

Appendix Figure S1. Kif23 is expressed in progenitors in the embryonic mouse cortex.

Appendix Figure S2. *Kif23* knockdown cortices contain pyknotic doublet neurons and progenitors.

Appendix Figure S3. Kif23 deficiency reduces cell proliferative capacity and mitotic activity.

Appendix Figure S4. Knockdown of *Kif23* causes the loss of apical centrosomes.

Appendix Figure S5. *Kif23* is expressed in the developing ferret cortex.

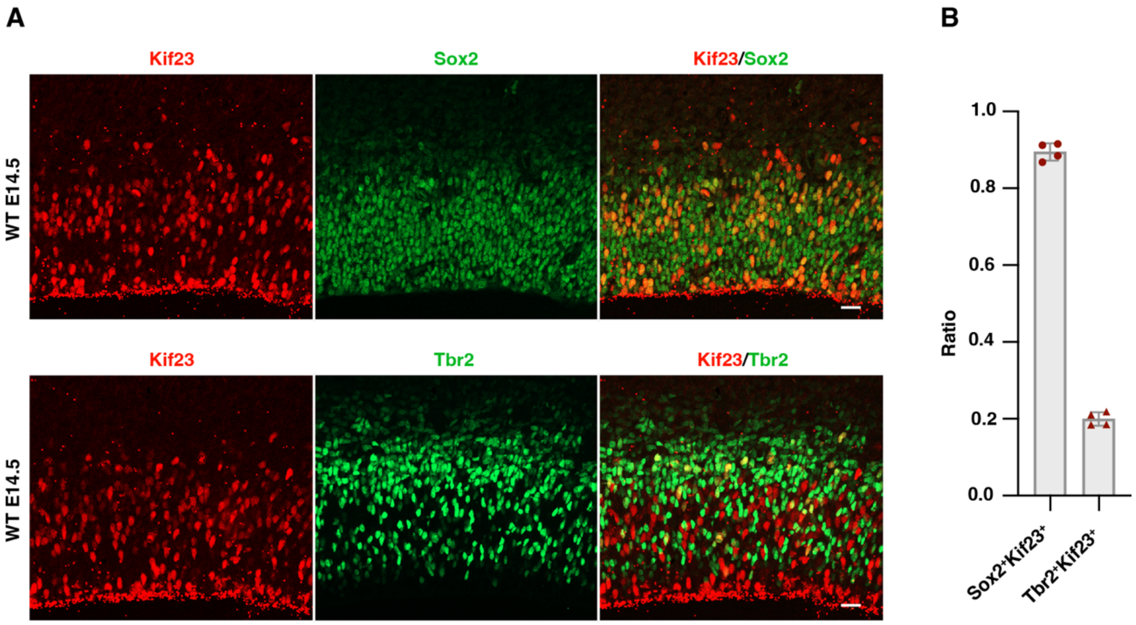

Appendix Fig S1. Kif23 is expressed in progenitors in the embryonic mouse cortex.

(A) Representative images of E14.5 mouse cortices stained for Kif23 and Sox2 or Kif23 and Tbr2**.** Scale bars, 20 µm

(B) Quantification of the ratio of $Kif23^{+}Sox2^{+}$ cells or $Kif23^{+}Tbr2^{+}$ cells relative to the total $Kif23^{+}$cells within the 300 µm wide column of E14.5 cortices. The data represent the mean ± SD (n = 815 cells, 4 embryos for $Kif23^{+}Sox2^{+}$ group; n = 784 cells, 4 embryos for $Kif23^{+}Tbr2^{+}$ group).

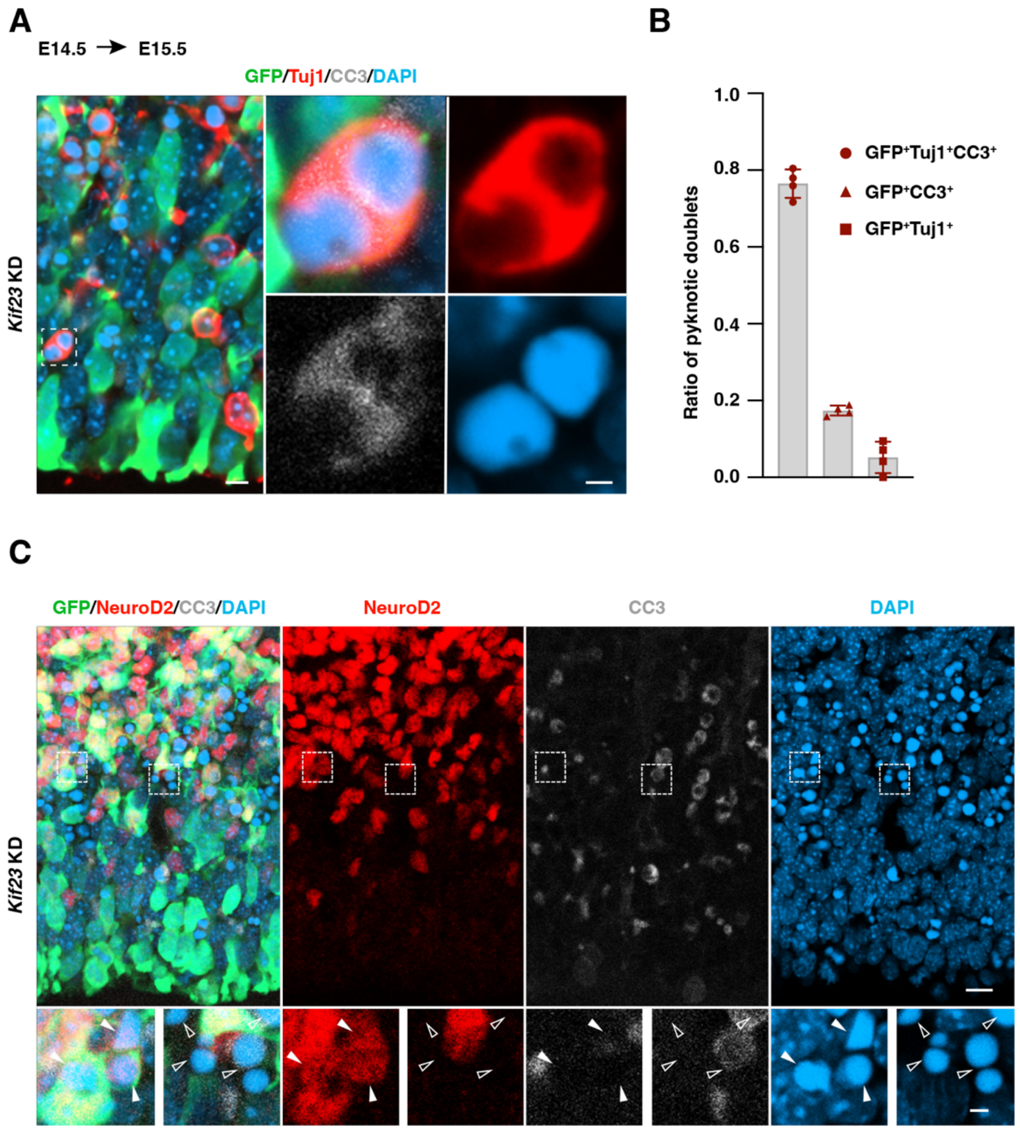

Appendix Figure S2. *Kif23* knockdown cortices contain pyknotic doublet neurons and progenitors.

(A, B) Representative images of E15.5 *Kif23*-KD cortical section stained for GFP, CC3, Tuj1, and DAPI **(A)**. Boxed area denotes zoomed areas. Scale bars, 5 μm (left); 1 μm (right). Quantification of the ratio of pyknotic doublet cells positive for GFP, CC3, and Tuj1 **(B)**. The data represent the mean ± SD. (*Kif23*-KD n = 103 cells, 4 embryos).

(C) Representative images of E15.5 *Kif23*-KD cortical section stained for GFP, CC3, NeuroD2, and DAPI. Boxed areas denote zoomed areas. Closed and open arrowheads indicate NeuroD2^{+} and NeuroD2^{-} pyknotic cells, respectively. Scale bars, 10 μm (top); 2 μm (bottom).

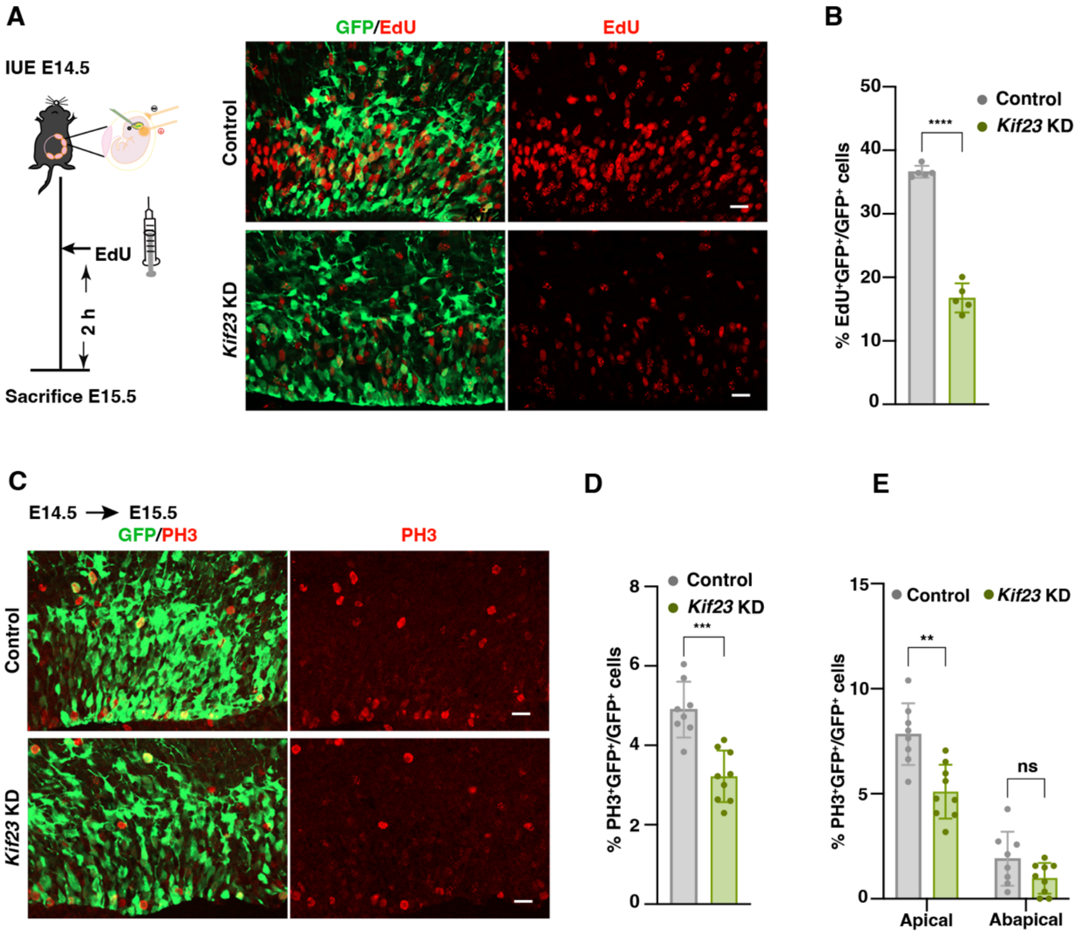

Appendix Figure S3. Kif23 deficiency reduces cell proliferative capacity and mitotic activity.

(A, B) Timeline of the *in-utero* electroporation and EdU injection (top). Electroporated cortical sections were stained for GFP and EdU (bottom) **(A)**. Scale bars, 20 µm. Quantification of the percentage of $EdU^{+}GFP^{+}$ cells relative to the total GFP^{+} cells within the 300 µm wide column of E15.5 cortices **(B)**. The data represent the mean ± SD (Control n = 2458 cells, 5 embryos; *Kif23*-KD n = 2141 cells, 5 embryos.). Two-tailed Student's *t*-test, ****p = 9.3E-08.

(C, D, E) Representative images of the control and *Kif23*-KD cortices at E15.5 stained for GFP and PH3. Scale bars, 20 µm **(C)**. Quantification of the percentage of $PH3^{+}GFP^{+}$ cells relative to the total GFP^{+} cells within the 300 µm wide column of E15.5 cortices. The data represent the mean ± SD (Control, n = 3737 cells, 8 embryos; *Kif23*-KD n = 4138 cells, 9 embryos). Two-tailed Student's *t*-test, ***p = 0.000122 **(D)**;Multiple *t*-tests, **p = 0.001880; ns, not significant **(E)**.

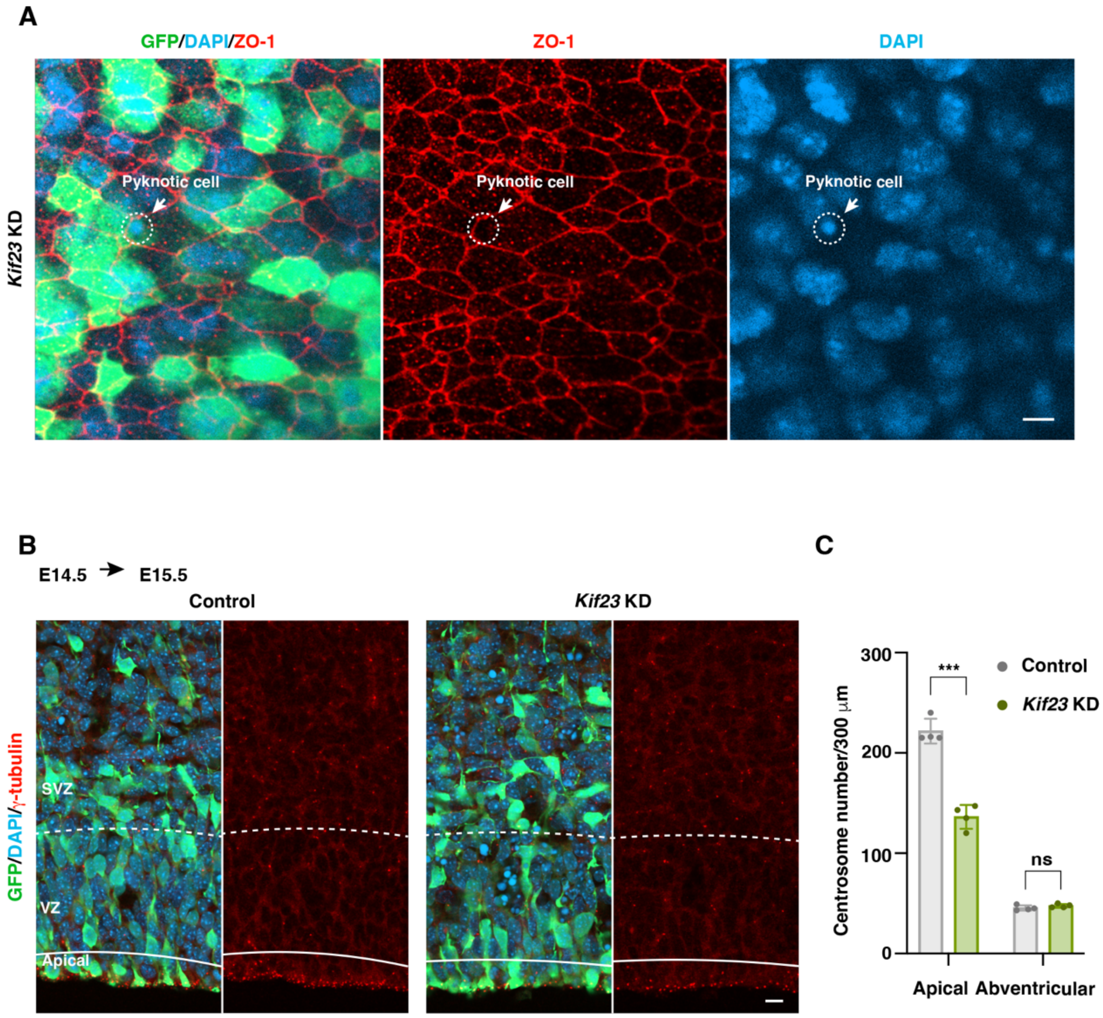

Appendix Figure S4. Knockdown of *Kif23* causes the loss of apical centrosomes.

(A) Whole-mount image of the electroporated brain section immunostained for GFP, ZO-1, and DAPI. The dotted circle marks the apoptotic cell. Scale bar 5 µm.

(B, C) Representative images of E15.5 control and *Kif23*-KD cortices stained for GFP, γ-tubulin, and DAPI **(B)**. The solid line delineates the boundary between apical surface and VZ; the dashed line delineates the boundary between VZ and SVZ. Scale bar, 10 µm. Quantification of the number of centrosomes in the apical surface and the VZ within the 350 µm wide column of E15.5 cortices **(C).** The data represent the mean ± SD (Control n = 4 embryos; *Kif23*-KD n = 4 embryos). Multiple *t*-tests, ***p = 0.000121; ns, not significant.

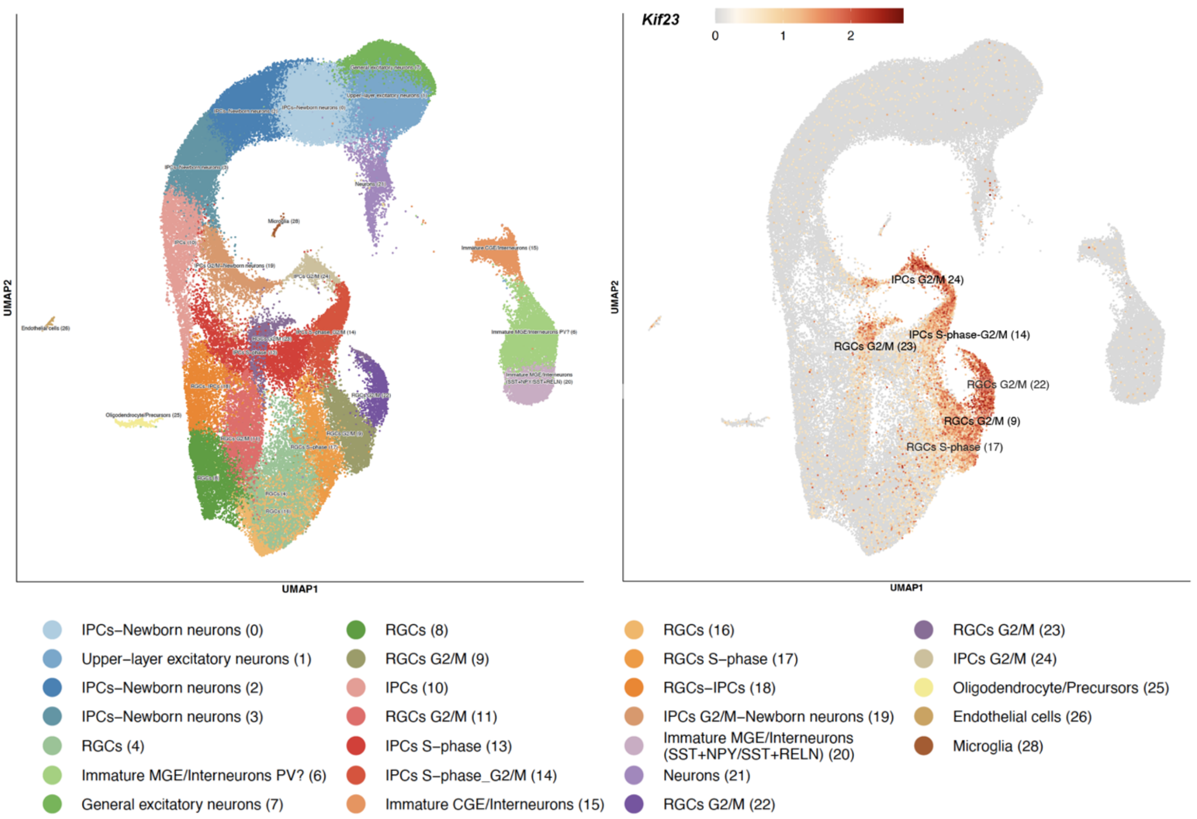
Kif23
0
1
2
UMAP2
UMAP1
IPCs G2/M 24)
IPCs S-phase-G2/M (14)
RGCs G2/M (23)
RGCs G2/M (22)
RGCs G2/M (9)
RGCs S-phase (17)
IPCs–Newborn neurons (0)
Upper–layer excitatory neurons (1)
IPCs–Newborn neurons (2)
IPCs–Newborn neurons (3)
RGCs (4)
Immature MGE/Interneurons PV? (6)
General excitatory neurons (7)
RGCs (8)
RGCs G2/M (9)
IPCs (10)
RGCs G2/M (11)
IPCs S–phase (13)
IPCs S–phase_G2/M (14)
Immature CGE/Interneurons (15)
RGCs (16)
RGCs S–phase (17)
RGCs–IPCs (18)
IPCs G2/M–Newborn neurons (19)
Immature MGE/Interneurons (SST+NPY/SST+RELN) (20)
Neurons (21)
RGCs G2/M (22)
RGCs G2/M (23)
IPCs G2/M (24)
Oligodendrocyte/Precursors (25)
Endothelial cells (26)
Microglia (28)

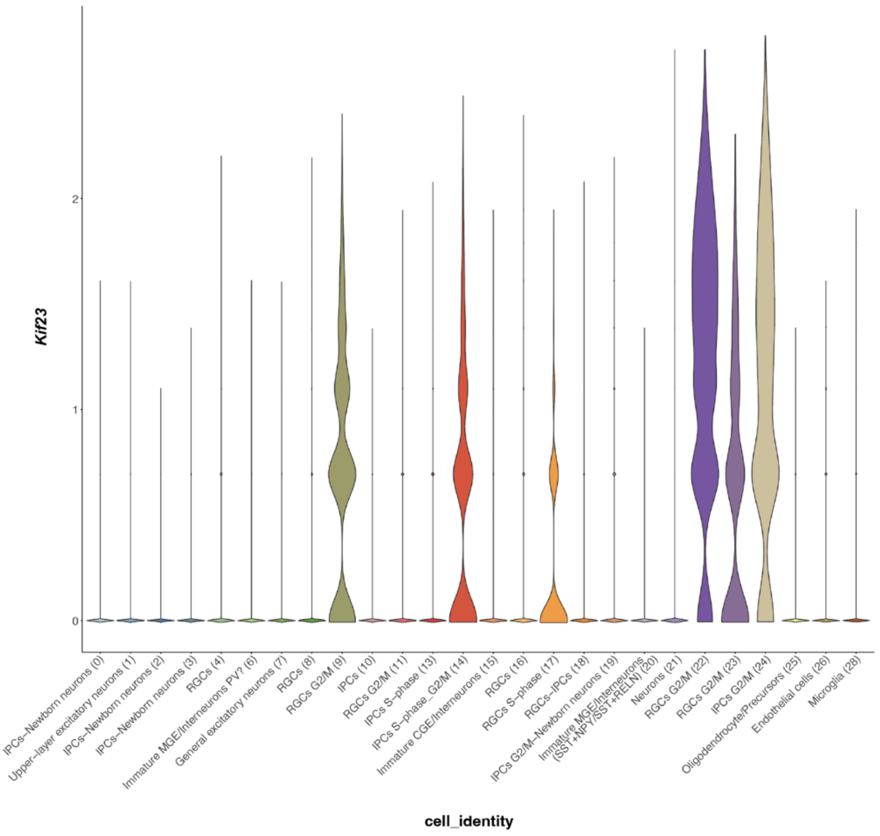
Kif23
0
1
2
cell_identity

Appendix Figure S5. *Kif23* is expressed in th developing ferret cortex.

Single-cell RNA-seq analysis of the developing ferret cortical germinal zones at embryonic day 34 and postnatal day 1(Del-Valle-Anton et al., 2024) showing cell type clusters (top-left), feature plots (top-right), and violin plots (bottom) to show the expression of *Kif23* in distinct RGC and IPC clusters in S/G2/M phase.
