# Supplementary figures and images for: Kinesin-like motor protein KIF23 maintains neural stem and progenitor cell pools in the developing cortex
Source: EMBO J. 2024 Dec 4;44(2):331–55. doi: 10.1038/s44318-024-00327-7 (PMC11729872; doi:10.1038/s44318-024-00327-7)

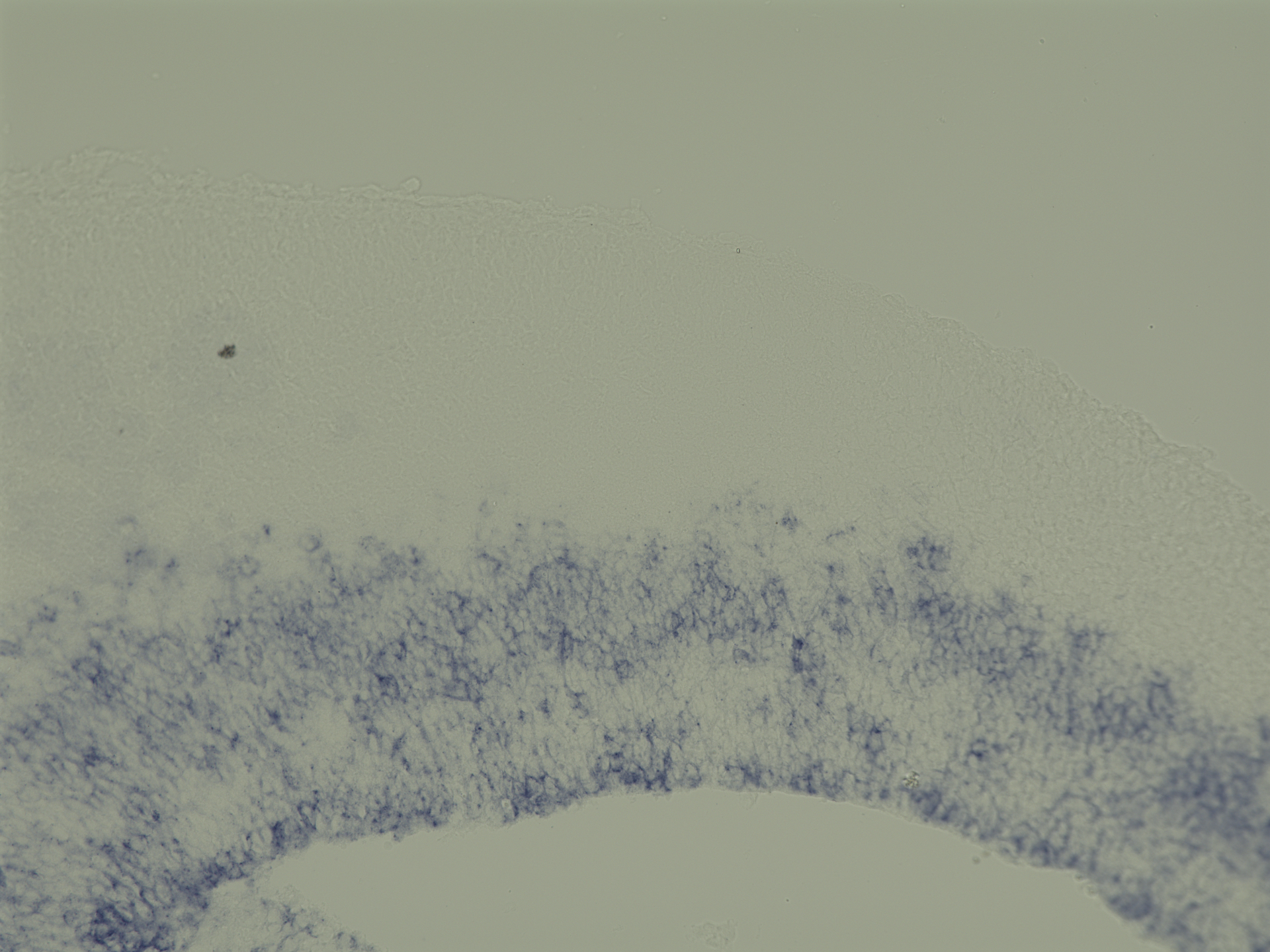

Supplement: Supplementary file 3 — Source data Fig. 1 [file 44318_2024_327_MOESM3_ESM.zip › EMBOJ-2024-117529R_ SourceDataFORFigure1/Fig 1C_image data/image_Kif23_ISH.tif]

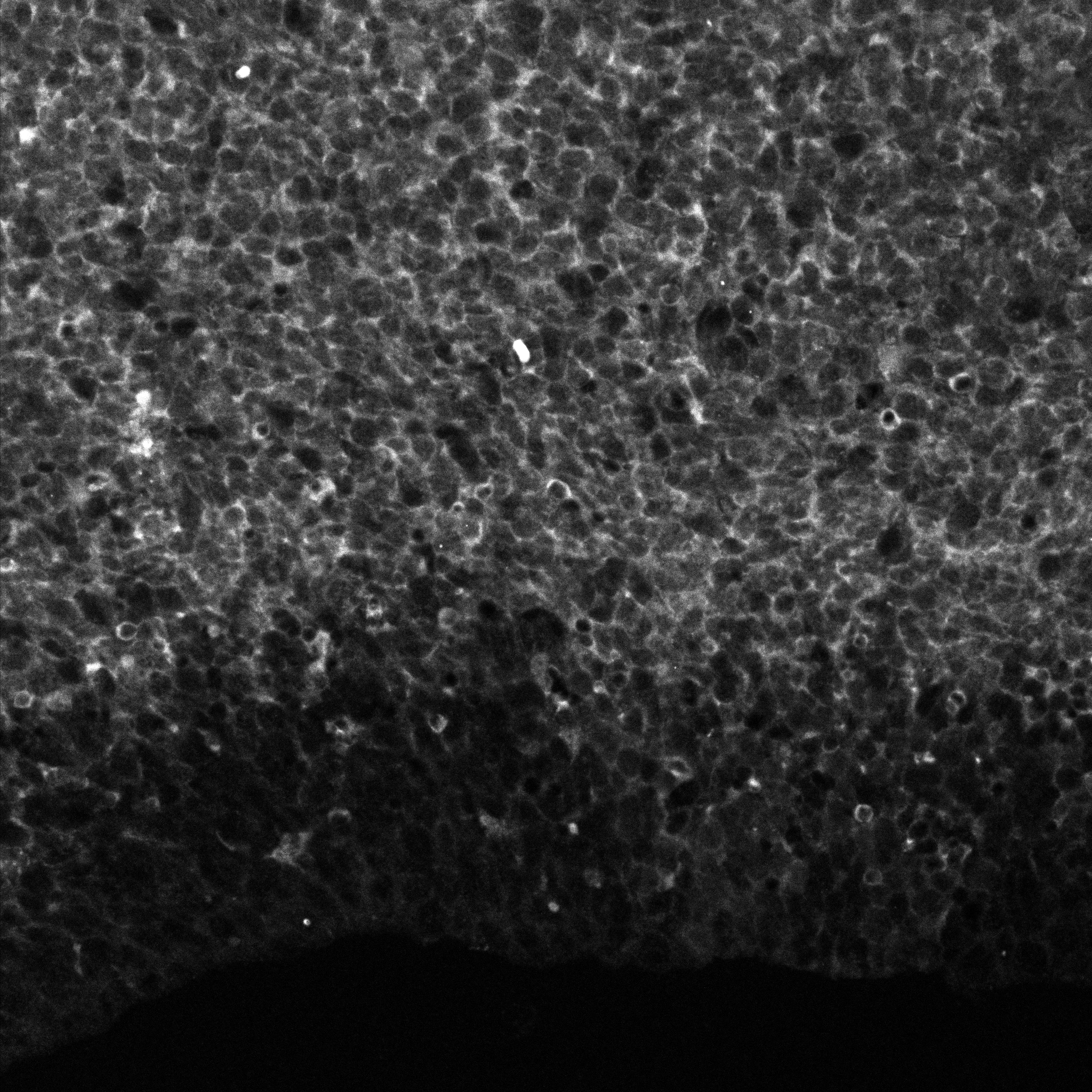

Supplement: Supplementary file 5 — Source data Fig. 3 [file 44318_2024_327_MOESM5_ESM.zip › EMBOJ-2024-117529R_ SourceDataFORFigure3/Fig 3D_image data/Fig 3D_Kif23 KD_GFP:Hu:DAPI.tif]

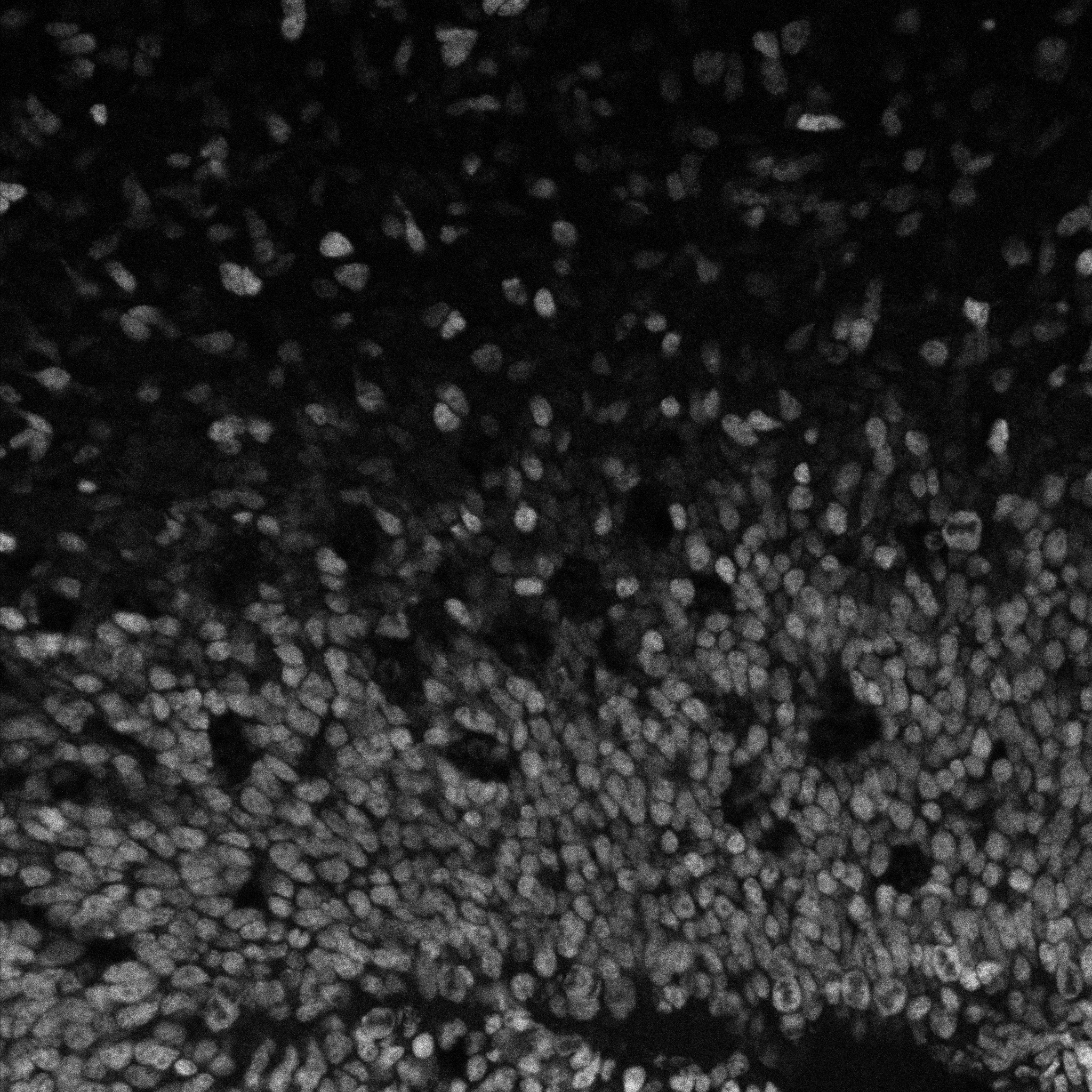

Supplement: Supplementary file 5 — Source data Fig. 3 [file 44318_2024_327_MOESM5_ESM.zip › EMBOJ-2024-117529R_ SourceDataFORFigure3/Fig 3D_image data/Fig 3D_Kif23 KD_GFP:Sox2:DAPI.tif]

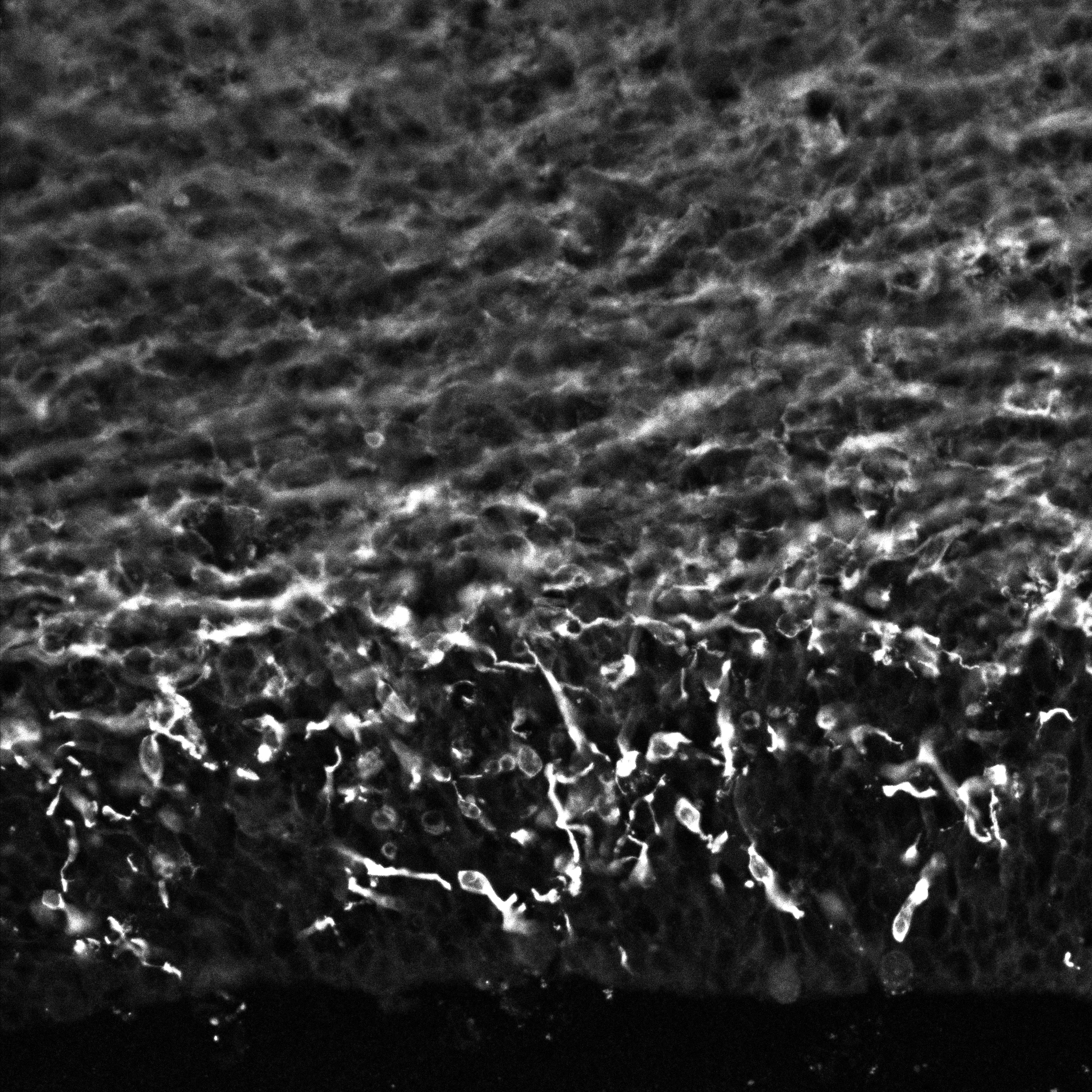

Supplement: Supplementary file 5 — Source data Fig. 3 [file 44318_2024_327_MOESM5_ESM.zip › EMBOJ-2024-117529R_ SourceDataFORFigure3/Fig 3D_image data/Fig 3D_Kif23 KD_GFP:Tuj1:DAPI.tif]

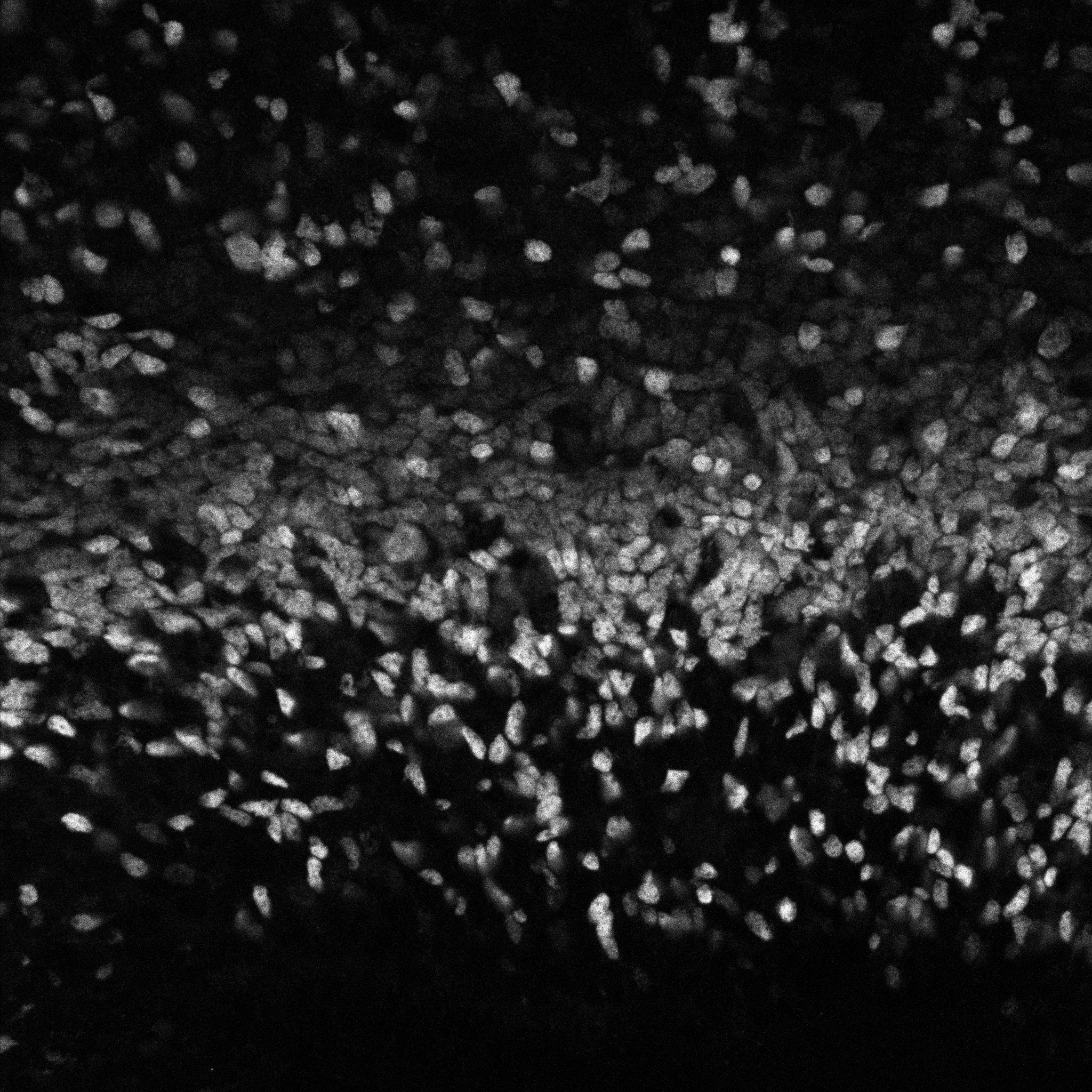

Supplement: Supplementary file 5 — Source data Fig. 3 [file 44318_2024_327_MOESM5_ESM.zip › EMBOJ-2024-117529R_ SourceDataFORFigure3/Fig 3D_image data/Fig 3D_Kif23 KD_GFP:Tbr2:DAPI.tif]

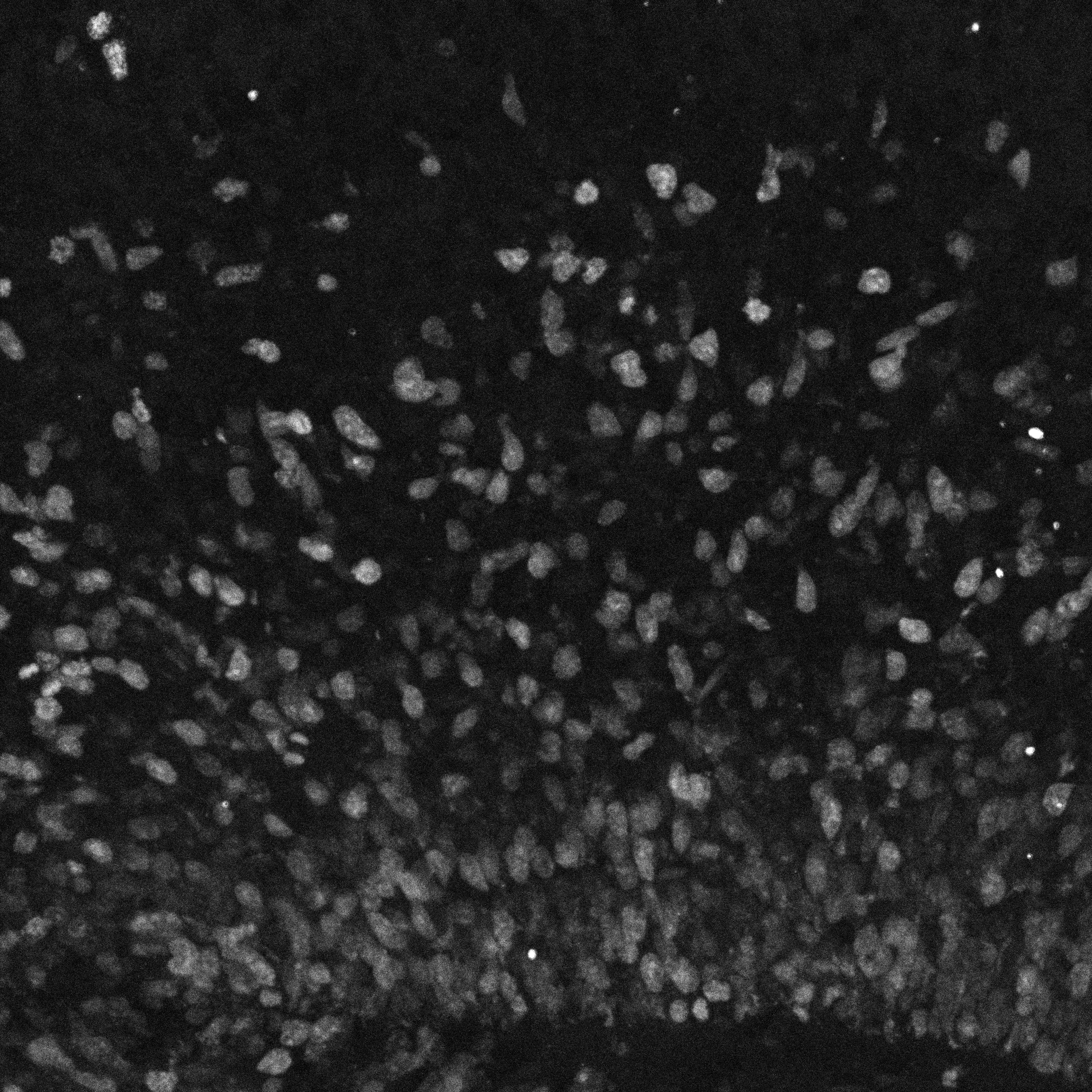

Supplement: Supplementary file 6 — Source data Fig. 4 [file 44318_2024_327_MOESM6_ESM.zip › EMBOJ-2024-117529R_ SourceDataFORFigure4/Fig 4I_Image data/Fig 4I_Control_GFP:EdU:Ki67.tif]

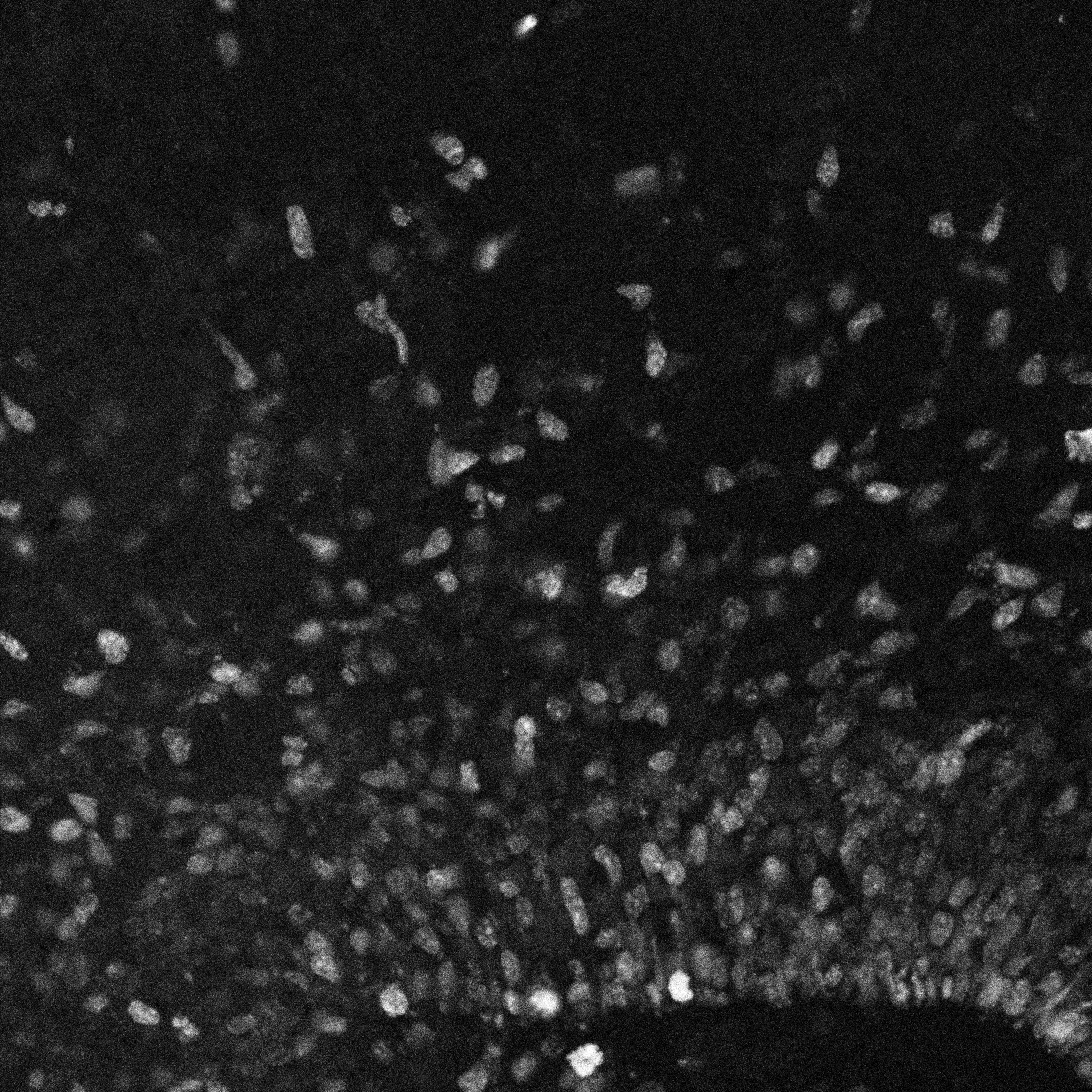

Supplement: Supplementary file 6 — Source data Fig. 4 [file 44318_2024_327_MOESM6_ESM.zip › EMBOJ-2024-117529R_ SourceDataFORFigure4/Fig 4I_Image data/Fig 4I_Kif23 KD_GFP:EdU:Ki67.tif]

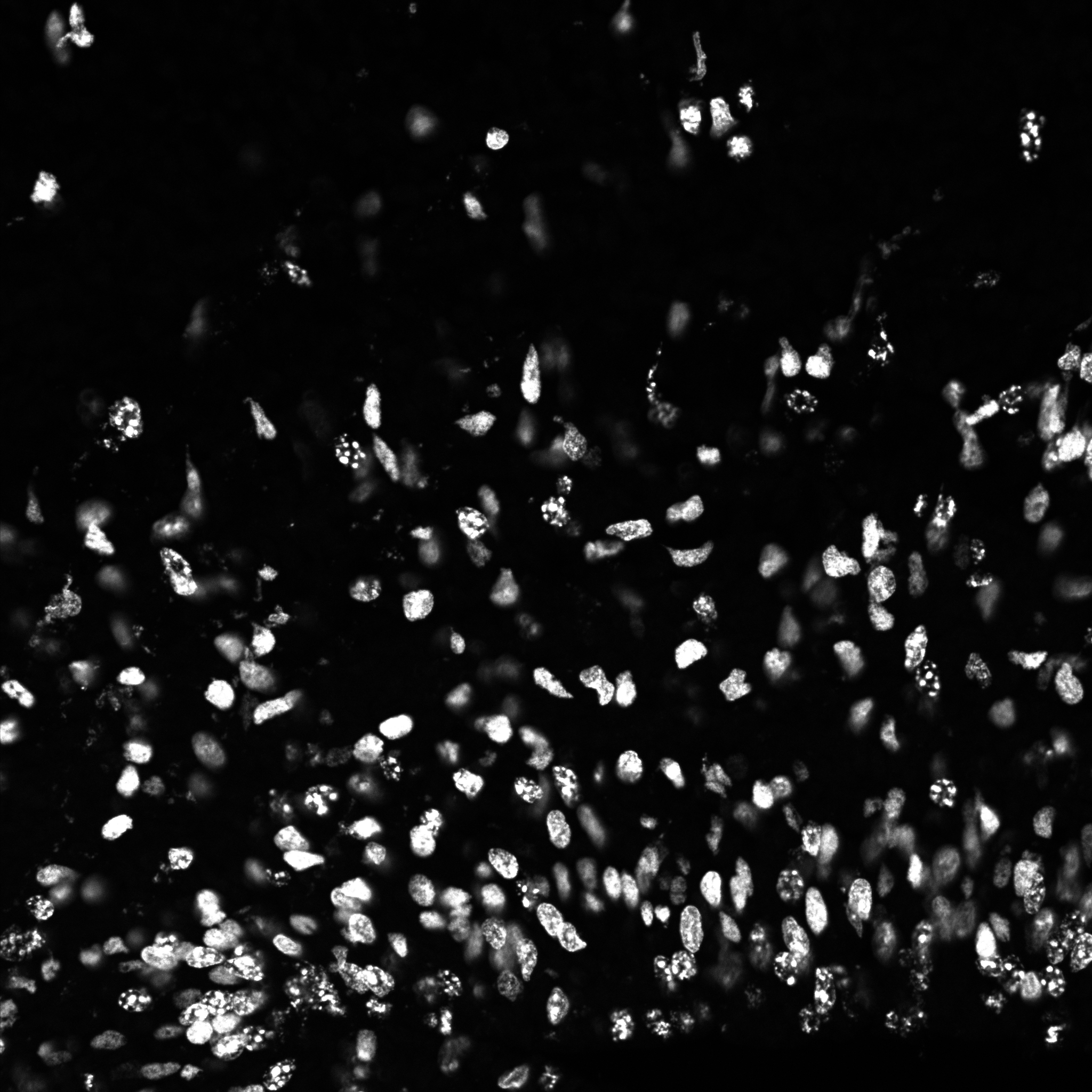

Supplement: Supplementary file 6 — Source data Fig. 4 [file 44318_2024_327_MOESM6_ESM.zip › EMBOJ-2024-117529R_ SourceDataFORFigure4/Fig 4E_Image data/Fig 4E_Control_GFP:EdU.tif]

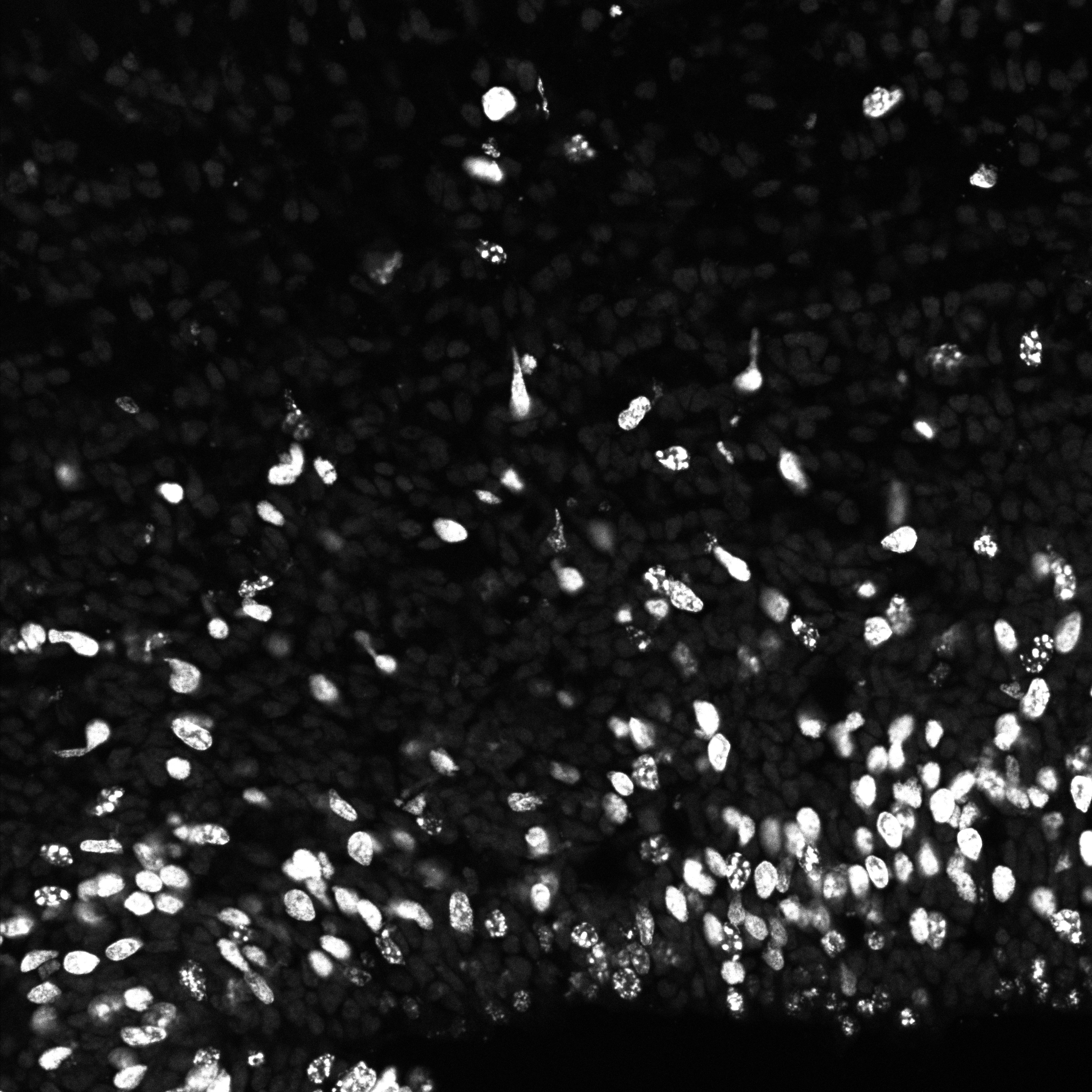

Supplement: Supplementary file 6 — Source data Fig. 4 [file 44318_2024_327_MOESM6_ESM.zip › EMBOJ-2024-117529R_ SourceDataFORFigure4/Fig 4E_Image data/Fig 4E_Kif23 KD_GFP:EdU.tif]

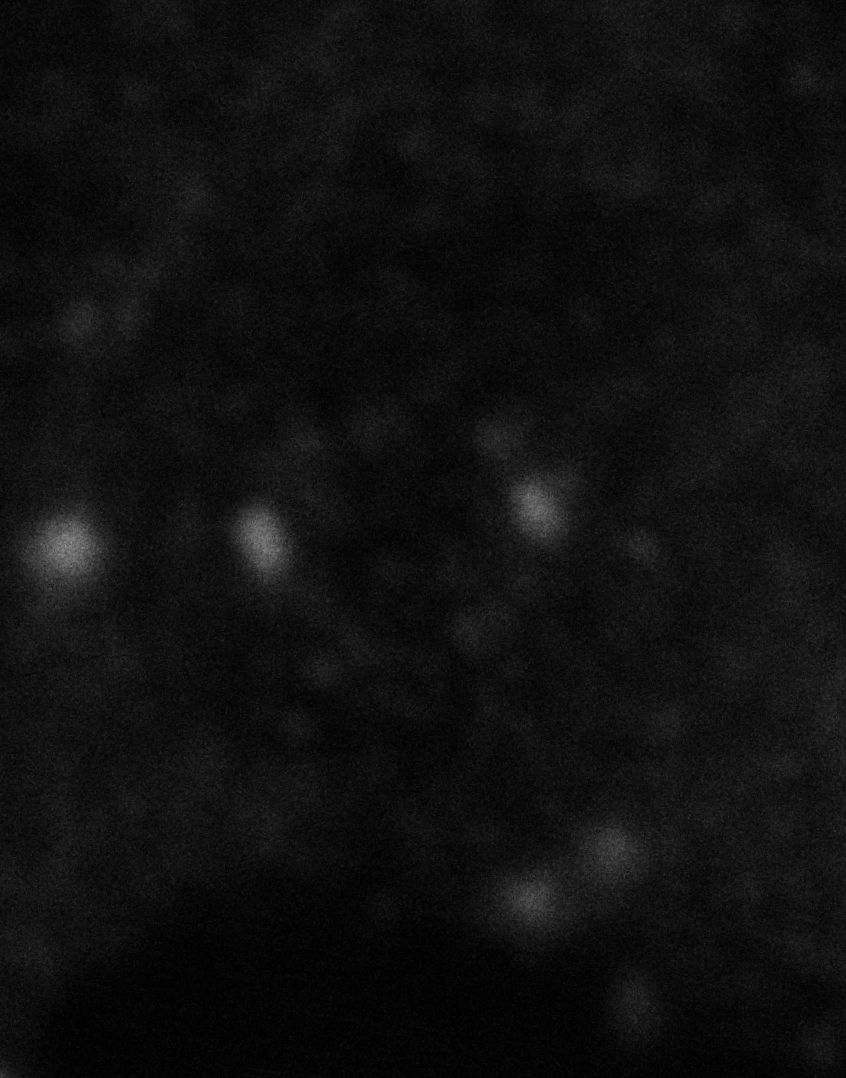

Supplement: Supplementary file 7 — Source data Fig. 5 [file 44318_2024_327_MOESM7_ESM.zip › EMBOJ-2024-117529R_ SourceDataFORFigure5/Fig 5C_Image data/Fig 5C_Control_GFP:alpha tubulin:gamma tubulin:DAPI.tif]

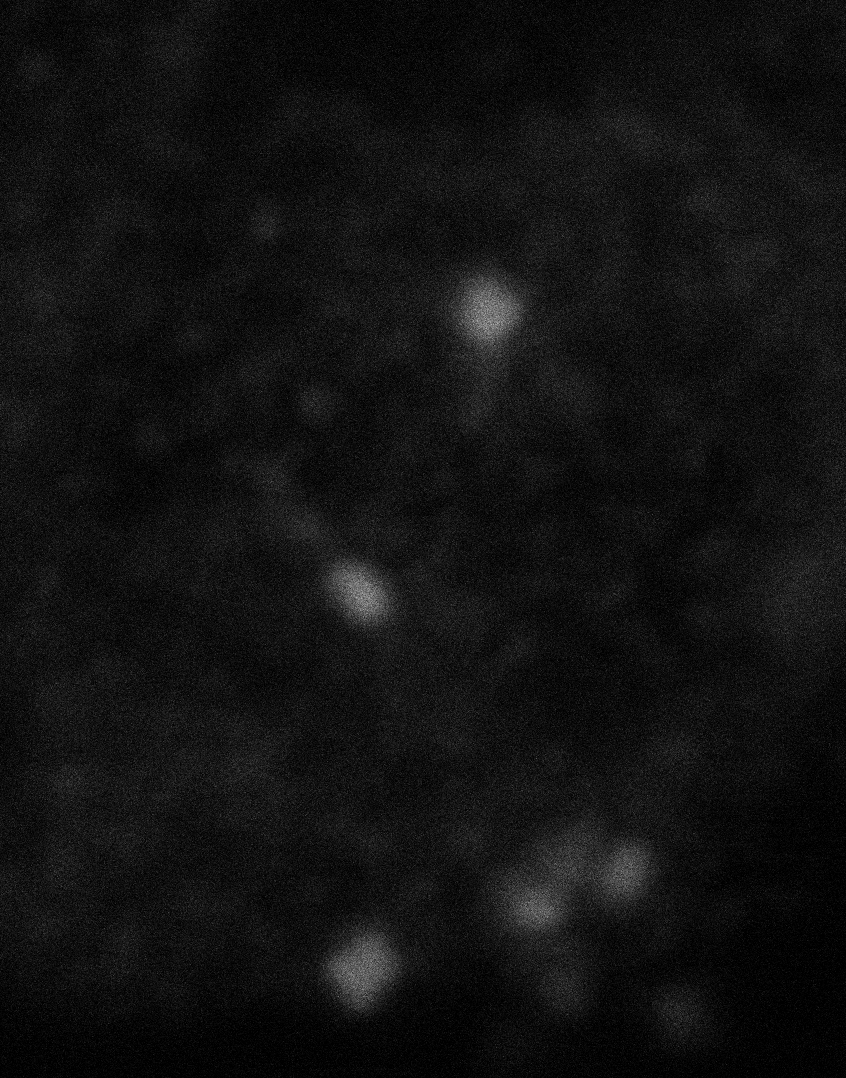

Supplement: Supplementary file 7 — Source data Fig. 5 [file 44318_2024_327_MOESM7_ESM.zip › EMBOJ-2024-117529R_ SourceDataFORFigure5/Fig 5C_Image data/Fig 5C_Kif23 KD_GFP:alpha tubulin:gamma tubulin:DAPI.tif]

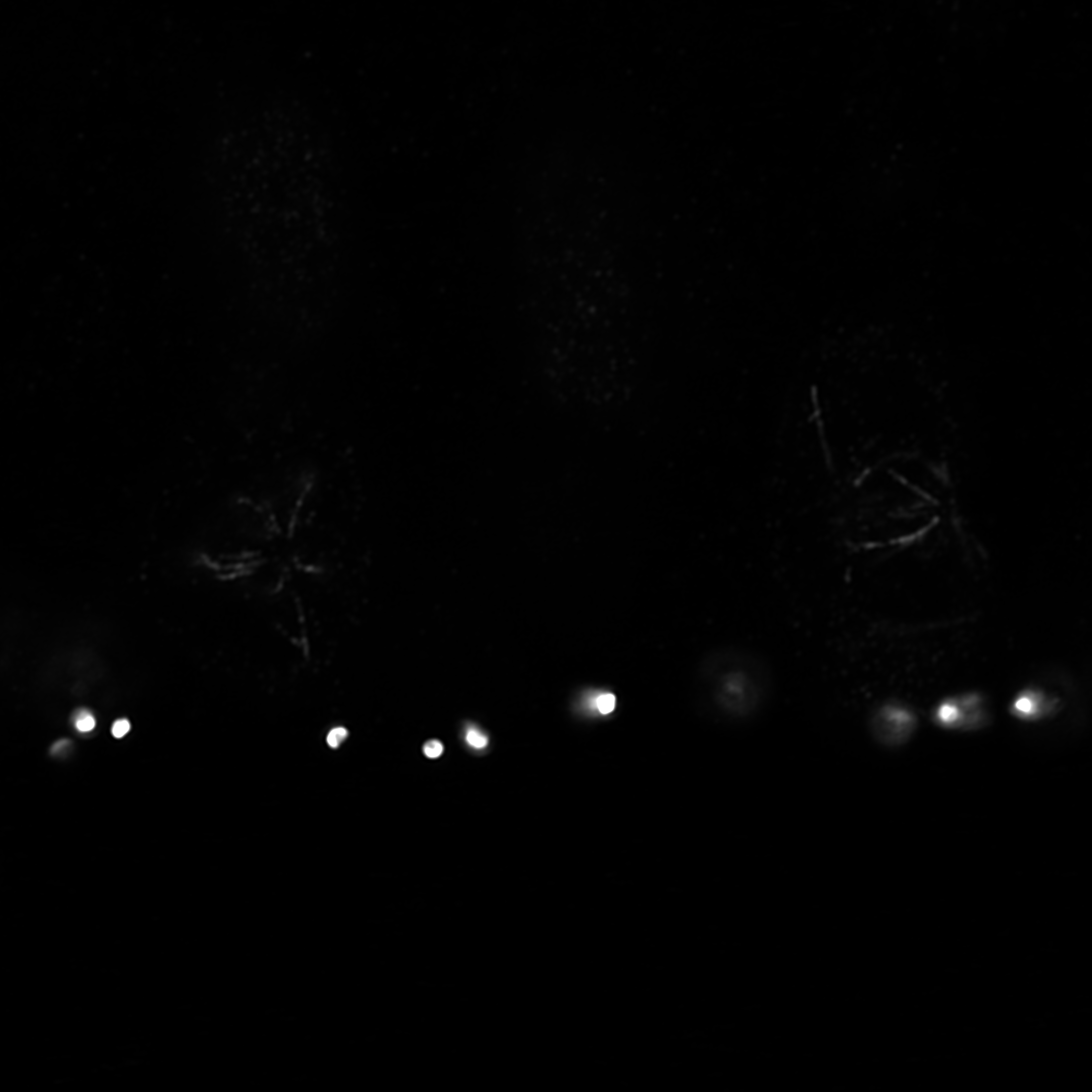

Supplement: Supplementary file 8 — Source data Fig. 6 [file 44318_2024_327_MOESM8_ESM.zip › EMBOJ-2024-117529R_ SourceDataFORFigure6/Fig 6E_Image data/Fig 6E_WT_Kif23:Cit-K.tif]
