# Supplementary material for: Kinesin-like motor protein KIF23 maintains neural stem and progenitor cell pools in the developing cortex
Source: EMBO J. 2024 Dec 4;44(2):331–55. doi: 10.1038/s44318-024-00327-7 (PMC11729872; doi:10.1038/s44318-024-00327-7)
Supplement: Supplementary file 12 — Expanded View Figures [file 44318_2024_327_MOESM12_ESM.pdf]

Expanded View Figures

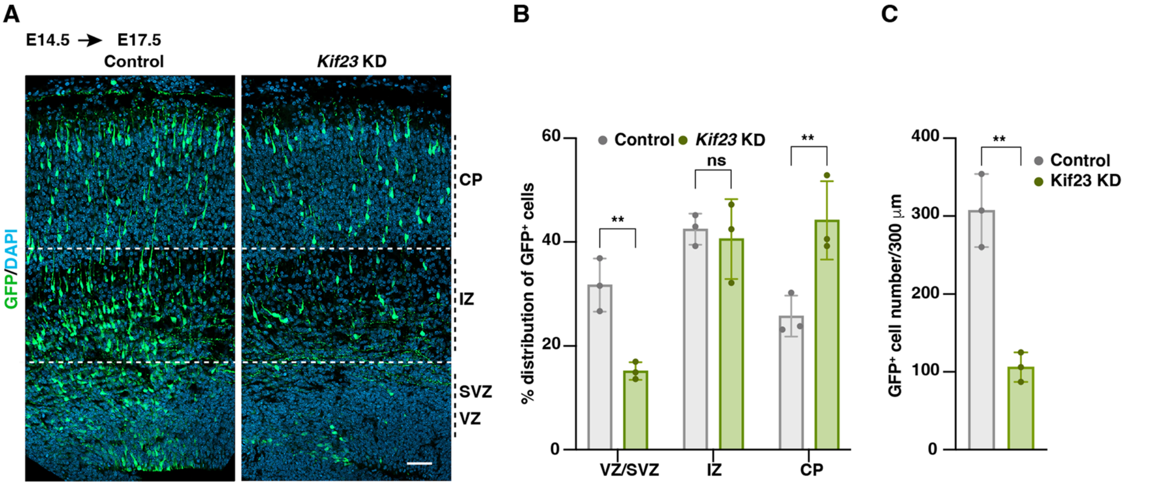

Figure EV1. Knockdown of *Kif23* leads to profound loss of GFP expressing cells.

(**A**) Representative images of GFP^+ cells distribution in the control and *Kif23*-KD cortices at E17.5. Dashed lines illustrate the borders among VZ/SVZ, IZ, and CP. Scale bar, 50 µm. (**B**) Quantification of GFP^+ cells distribution in VZ/SVZ, IZ, and CP, respectively within the 300 µm wide column of E17.5 cortices. The data represent the mean ± SD Control $n = 921$ cells, 3 embryos; *Kif23*-KD $n = 318$ cells, 3 embryos). Two-way ANOVA with Bonferroni's multiple comparison test, *p* values from left to right: $^{**}p = 0.007258$, $^{**}p = 0.003357$, ns: not significant. (**C**) Quantification of the average number of GFP^+ cells within the 300 µm wide column of E17.5 cortices. The data represent the mean ± SD (Control $n = 921$ cells, 3 embryos; *Kif23*-KD n = 318 cells, 3 embryos). Two-tailed Student's *t* test, $^{**}p = 0.002355$.

© The Author(s)

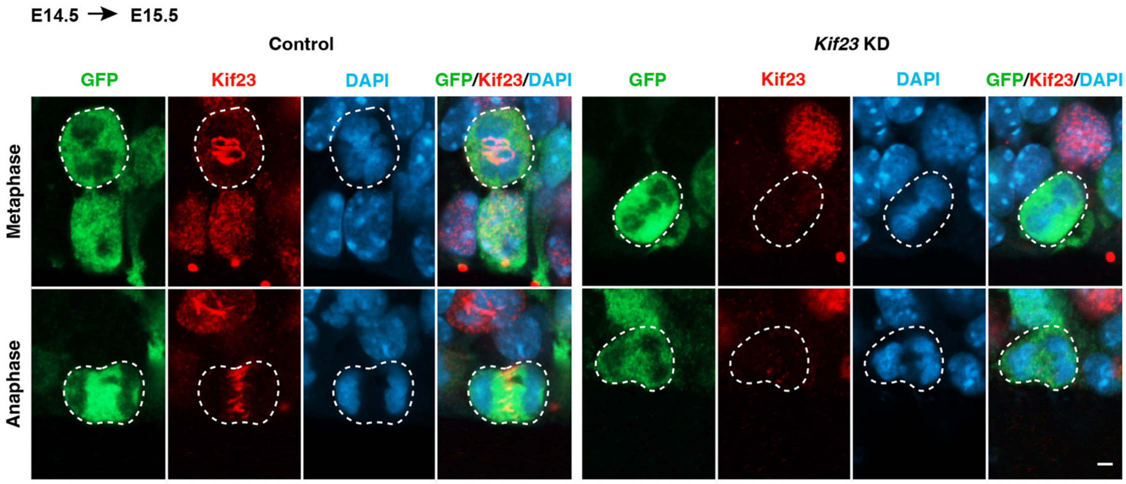

Figure EV2. *Kif23* knockdown reduces Kif23 protein level in the microtubule.

Representative images of the control and *Kif23*-KD cortical sections at E15.5 stained for GFP, Kif23, and DAPI. Examples of GFP^+ cells at the metaphase or anaphase stage are outlined. Scale bar, 2 µm.

 © The Author(s)

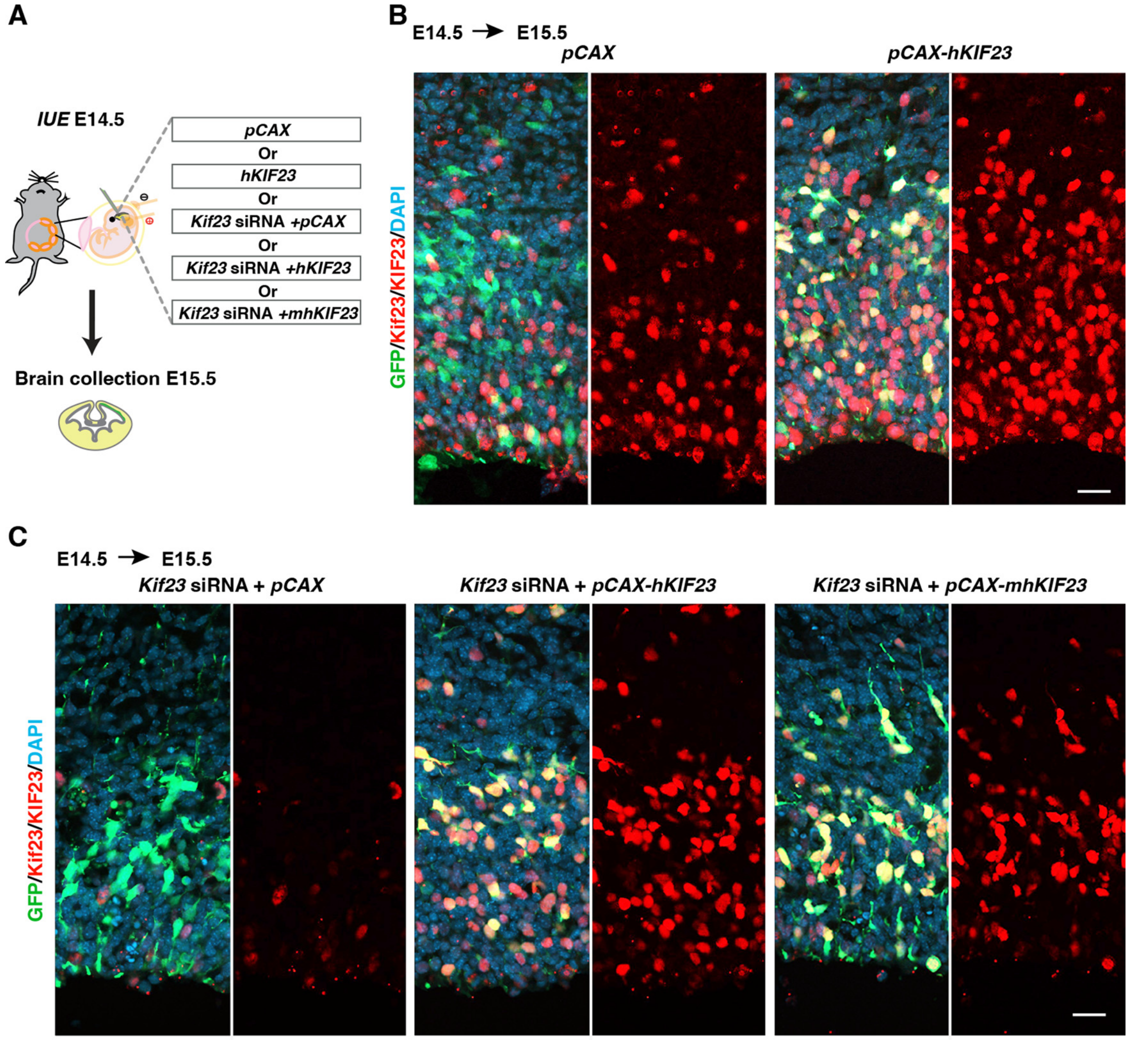

Figure EV3. ***Kif23* siRNA decreases mouse Kif23 protein levels, not human KIF23.**

(**A**) Schematic overview of the experiment. (**B**) Representative images of mouse cortices one day after electroporation of *pCAX* or *pCAX-hKIF23* stained for GFP and Kif23/KIF23. Scale bar, 20 μm. (**C**) Representative images of mouse cortices one day after electroporation of *Kif23* siRNA/*pCAX*, *Kif23* siRNA/*pCAX-hKIF23* or *Kif23* siRNA/*pCAX-mhKIF23* stained for GFP and Kif23/KIF23. Scale bar, 20 μm.
